# Fully inkjet-printed flexible organic voltage inverters as a basic component in digital NOT gates

**DOI:** 10.1038/s41598-022-14797-4

**Published:** 2022-06-28

**Authors:** Adam Luczak, Kalyan Y. Mitra, Reinhard R. Baumann, Ralf Zichner, Beata Luszczynska, Jaroslaw Jung

**Affiliations:** 1 Department of Molecular Physics, Faculty of Chemistry, Lodz University of Technology, Zeromskiego 116, 90-924 Lodz, Poland; 2 Faculty of Mechanical Engineering, Institute for Print and Media Technology, Technische Universität Chemnitz, Reichenhainer Strasse 70, 09126 Chemnitz, Germany; 3 Department Printed Functionalities, Fraunhofer Institute for Electronic Nano Systems ENAS, Technologie-Campus 3, 09126 Chemnitz, Germany

**Keywords:** Electronic devices, Electronic devices, Molecular electronics, Electronic properties and materials

## Abstract

In relation to conventional vacuum-based processing techniques inkjet printing enables upscaling fabrication of basic electronic elements, such as transistors and diodes. We present the fully inkjet printed flexible electronic circuits, including organic voltage inverter which can work as a NOT logic gate. For this purpose the special ink compositions were formulated to preparation of gate dielectric layer containing poly (4-vinylphenol) and of the semiconductor layer poly[2,5-(2-octyldodecyl)-3,6-diketopyrrolopyrrole-alt-5,5-(2,5-di(thien-2-yl)thieno [3,2-b]thiophene)]. A printed photoxidized poly (3-hexyltiophene) semiconductor was used as the active layer of the resistors. The operation of the printed inverters and NOT logic gates was analyzed based on the DC current–voltage characteristics of the devices. The resistance of the devices to atmospheric air was also tested. Not encapsulated samples stored for three years under ambient conditions. Followed by annealing to remove moisture showed unchanged electrical parameters in comparison to freshly printed samples.

## Introduction

Inkjet printing is one of the most promising methods of manufacturing organic electronic devices. Because the printed pattern can be changed easily, this method is perfectly suited to developing prototypes and small in-house electronic devices. Similarly to 3D printers, inkjet printers can be used for manufacturing organic electronics on an industrial scale, but also in homes and workshops to prepare small electronic projects. Inkjet printing does not require high temperatures, a vacuum, lithography, or other costly subtractive methods^1^. Moreover, electronic elements can be produced on flexible, transparent foils^2,3^. First, however, the ink formula and printing parameters must be optimized^4–6^.

All-inkjet printed organic thin film transistors (OTFT) have been manufactured for many years^7–12^. Usually the electrical parameters of printed OTFTs are generally worse than those of transistors produced by conventional techniques. However, the main problem with long term stability over time remains unresolved^13,14^. This is because inkjet printing provides less control over the morphology and material sophistication of the film^15^. These problems must be resolved in the laboratory before commercialization and practical implementation of inkjet printing can proceed in industrial and home applications. Printing more complicated electronic devices requires very precise research on the process of printing layers on the surfaces of previously printed layers^5,16,17^.

One of the simplest electronic elements that can perform a useful logic function is a voltage inverter operating as NOT logic gate. All-inkjet printed logic gates can be produced in two configurations: in circuits with a complementary pair of p-type and n-type transistors operating simultaneously^18^, or in a unipolar configuration where one transistor and one resistor work in a single voltage inverter. A transistor with a shorted gate and source electrodes is often used as a resistor^19,20^. In most previous research on printed logic gates, printing has been used to apply only one layer or some of the layers—e.g. electrodes and semiconductors. Other layers, such as a dielectric, have been deposited by other methods. The semiconducting layer is generally printed with commercially available inks based on organic semiconductors or carbon nanotubes^18,21,22^. All-inkjet printed devices are still rare, and one of the key reason is that inkjet printing technology is still insufficiently mastered. There are relatively few reports in the literature of NOT gates in which all the components, such as electrodes, current-conducting paths, and dielectric and semiconductor layers, were fabricated entirely using the inkjet printing technique. Fully printed logic gates with layers of semiconductors applied to a substrate by inkjet printing with a TIPS-pentacene solution are described in Refs.^18,23^. The resistors in the logic gates were obtained by connecting the gate and the source electrodes of the transistors. For such logic gates to function properly, their resistance should be much higher than the channel resistance in the on-state and much lower than the resistance of the transistor in the off-state. Some reports show that printing resistors with high resistance is difficult. Jung et al.^24^ described the properties of resistors fabricated by inkjet printing using a mixture of poly (3,4-ethylene-1,4-dioxythiophene) and sulfonated polystyrene called PEDOT:PSS. The resistors were printed in the form of a PEDOT:PSS line connecting two silver electrodes. This was achieved by reducing the concentration of the polymer and increasing the spacing between the droplets in the printed layer (low thickness). Printing multiple layers on top of each other allowed for a slight decrease in the resistance of the resulting resistor. These strategies made it possible to adjust its resistance according to requirements.

Here, we investigate an all-inkjet printed inverter working as an element in NOT logic gates in the unipolar configuration, with one transistor and one resistor in the structure. To produce paths and electrodes the commercially available silver ink was used. New ink formulas based on semiconductor and dielectric materials were elaborated in the laboratory. The ink compositions were optimized to simplify the printing process. Organic thin film transistors were printed in the bottom gate bottom contacts (BGBC) architecture, with poly (4-vinylphenol) as the gate dielectric and poly[2,5-(2-octyldodecyl)-3,6-diketopyrrolopyrrole-alt-5,5-(2,5-di(thien-2-yl)thieno [3,2-b]thiophene)] (DPPDTT) as a semiconductor. Photoxidized poly (3-hexyltiophene) (P3HT) was used as an active layer in the resistors. Photooxidation causes the transistors with poly (3-hexylthiophene) layers to lose their properties, so they are no longer sensitive to the electric field formed in the dielectric layer by the gate voltage^25^. The devices made with this photo-oxidized material showed ohmic current–voltage characteristics. The polymer could therefore be used as a resistor. Resistors were printed in the same way as the transistors, but without a gate electrode. To obtain a working inverter, the transistors and resistors were developed separately. The printing parameters i.e., the area of printed semiconductor layer, the thickness of the dielectric film, the dimensions of the transistor channel, etc. were optimized. On the basis of the DC current–voltage characteristics of the separately printed resistor and transistor, we prepared a model of the reference inverter. Graphical analysis of their operation was then performed. The parameters of the resistor and transistor were chosen to produce fully printed inverters with the best properties. The fully printable inverters were tested and analyzed to evaluate their performance.

In this work we propose the fully printed voltage inverter with one OTFT and one organic resistor. The important aspect of this devices is their thermal and time stability due to use of stable in air DPPDTT as the active OTFT layer and photooxidised P3HT. In most articles the authors present resistors made by conductors^1,7,24^. The high resistance is achieved by printing very small patterns or by incomplete sinterization of silver nanoparticles ink. Additionally the used process of printing resistors is much more repeatable and faster than printing of transistors so used in inverters instead of a resistor. There are several important problems associated with this method of fabrication, as e.g.: a weak repeatability of the process—in case of very small patterns, even a small printing error can significantly change the resistance value, low thermal stability—in resistors made of incompletely cross-linked ink, the resistor will turn into a conductor above 100 Celsius degrees. Ink containing P3HT are easily printed without the danger of nozzle clogging. Resistivity can be controlled through molecular mass and the printing area of P3HT. High resistivity can be achieved in as little as 4 mm^2^ (for a polymer with Mw = 30 kDa). The thermal and time stability of our devices is very high. Even after 3 years after the device has been manufactured, the resistor and transistor are still working. You may notice an increased turn-off current value in the OTFT, but after annealing process (in ambient atmosphere) the OTFT parameters were almost the same as just after printing.

We present a complete report on the fabrication of fully printed inverters, starting from their digital design, ink formulation based on selected chemical compounds, device construction, to the printing of stable and working devices. We also present a graphical analysis of the inverter, which is a novelty among organic electronic devices, considering its structure and fabrication method.

## Results and discussion

### Designing and analyzing all-inkjet printed inverters

**Figure 1:**
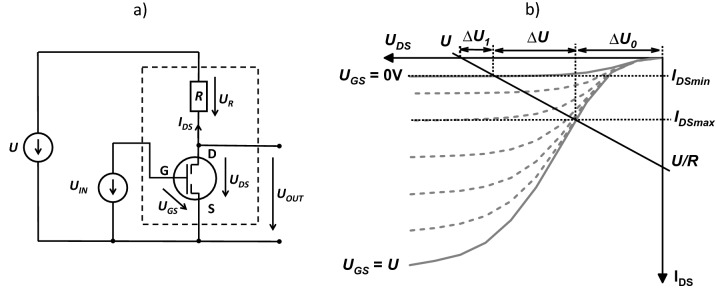
Schematic of the electrical circuit, including the tested voltage inverter (surrounded by dashed lines), power supply (*U*) and input voltage source (*U*_*IN*_) (**a**). Load line of the inverter with characteristic points of crossing with the axis (straight line), drawn on the output characteristic curve of the transistors (dashed and solid lines) (**b**). Dotted lines mark the maximum and minimum drain current (*I*_*DSmin*_*, I*_*DSmax*_) in the inverter and characteristic threshold voltages (Δ*U*_*1*_, Δ*U* and Δ*U*_*0*_).

The most effective approach to designing and testing the all-inkjet printed inverters was graphical analysis of their current–voltage characteristics. The graphical method requires experimental determination of the current–voltage output characteristics of the transistor and resistor. Using Kirchhoff’s second law and the Ohm law, for the resistor in the electric circuit loop shown in Fig. 1a we obtain:

1 $${U}_{DS}=U-{I}_{DS}R$$

Equation (1) defines the load line of the transistor. The load line passes through two characteristic points: the first is the supply voltage *U* marked on the abscissa axis and the second is the short drain and source current (*I* = *U*/*R*) on the ordinate axis. The load line was plotted on the same graph as the transistor current–voltage characteristic (see Fig. 1b).

The intersections of the load line with the OTFT output characteristic for *U*_*GS*_ = 0 V and *U*_*GS*_ = *U* correspond to the currents *I*_*DSmin*_ and *I*_*DSmax*_. Assuming that *U*_*IN*_ = *U*_*GS*_ and *U*_*OUT*_ = *U*_*DS*_, from Fig. 1a,b, and Eq. (1) it follows that

2 $$\begin{array}{l}{U}_{OUT,1}=U-{\Delta U}_{1}\;for\;{U}_{IN,0}=0 V\\ {U}_{OUT,0}={\Delta U}_{0}\;for\;{U}_{IN,1}=U\end{array}$$

where

3 $$\begin{array}{l}{\Delta U}_{1}={I}_{DSmin}R \\ {\Delta U}_{0}=U-{I}_{DSmax}R\end{array}$$

When the inverter is operating as a gate negator, we strive to ensure that the voltages Δ*U*_*0*_ and Δ*U*_*1*_ are as small as possible (the ratio Δ*U*/*U* was close to 1). Otherwise, voltages corresponding logic states “1” (high voltage) and “0” (low voltage) will not be sufficiently separated.

A number of processes were carried out to optimize the printing of individual layers of the device. Each layer of the conductors, semiconductors, and insulators required the development a specially formulated ink and optimization of the processes of printing and curing.

### Silver electrodes and conductive pads

**Figure 2:**
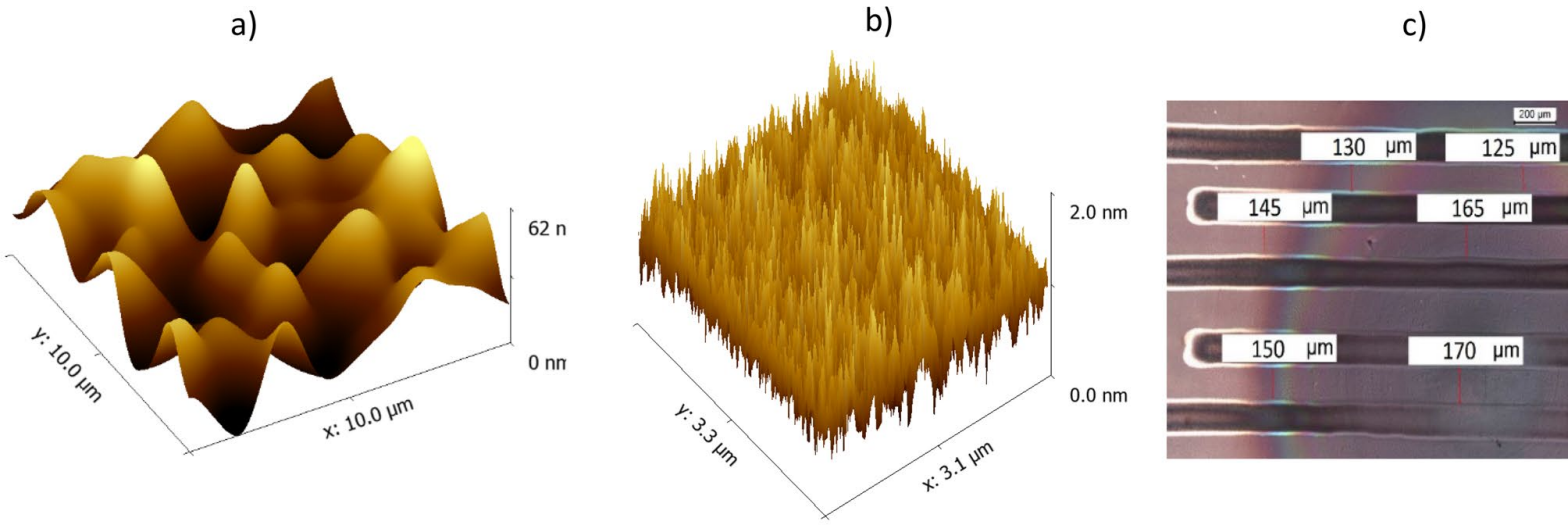
AFM image of the surface topology for a printed Ag conductive pad (**a**) and PVP dielectric layer (**b**). Optical microscope view of the semiconductor layers printed using optimized ink (the widths of the paths are indicated in the white boxes) (**c**).

The processes of printing and depositing the electrodes and conductive pads were optimized to provide a high-quality printed layer free of defects. Optimization involved changing the printing rate and drop volume, as well as the substrate temperature and the number of active nozzles in the printing head. The waveform controlling the piezoelectric element was tuned. As a result, geometrically continuous conductive silver pads and electrodes were obtained. The most optimal parameters for printing the devices were: 40 µm drop space; 40 °C substrate temperature; three functionally active and stable nozzles; 5 kHz jetting frequency. Analysis of the surface of the electrodes using the AFM technique showed that the electrode roughness was around 30 nm, which is relatively high considering its role as a bottom gate for OTFTs (Fig. 2a). For that reason, a dielectric layer printed on top of the gate electrode was required to provide additional planarization.

### Dielectric layer

The PVP dielectric was printed with a 20 µm drop space, on 25 °C substrate, using ten nozzles. It was finally obtained after printing two layers, wet-on-wet, with 5 kHz jetting frequency. The polymeric films were around 1000 nm thick. Analysis by AFM showed two different types of defects on the film surface: (1) agglomerates (large, light circles) and (2) pinholes (small, black circles) (see Fig. S1a in the supplementary materials). To remove the particle agglomerates, three-fold filtration of the ink was applied. Defects the in form of pinholes were considered as critical defects, because when printing S/D electrodes the Ag ink penetrated these holes, causing short circuits. To remove such defects, the printing process was run twice, without any intermediate curing of the first film. The layers printed using this method had thicknesses of around 1 µm and roughness of 1 nm (Fig. 2b). Thus, the high surface roughness of the Ag electrodes was not reproduced by the semiconductor layer. To check the dielectric properties of the printed films, Broadband Dielectric Spectroscopy was used. The relative permittivity was measured as a function of the sample temperature and frequency of the signal. Analysis of the real part of the permittivity (*ε*_*r*_) as a function of the signal frequency showed that the polymeric films had stable permittivity of around 3 across a wide range of frequencies (see Fig. S1b in the supplementary materials). This permittivity is typical for polymeric dielectrics. The dependence of *ε*_*r*_ on temperature shows that the relative permittivity remained constant up to 100 °C. This was very advantageous, as the printed layers of organic semiconductors could therefore be annealed at temperatures lower than 100 °C, avoiding deterioration of the properties of the transistors.

### Semiconductor layers

Two self-formulated inks containing DPPDTT and P3HT semiconductors were optimized to compare the electronic functionality of the samples. The viscosity of the ink was tuned with reference to its jetting behavior i.e., the shape and trajectory of the droplets generated by the DMC printhead of the DMP-2831 printer. Another comparison was also made to a commercially available ink, FS0096 by Flexink Ltd^26^. It was found that the use of toluene as a solvent in the P3HT- and DPPDTT-based inks did not allow for high-quality prints, despite correct drop generation by the printhead. The layers were heterogeneous and discontinuous, with many surface defects. The coffee stain effect was also very prominent (see Fig. S2 in the supplementary materials). The coffee stain effect was caused by the accumulation of the polymer on the edges of the drying droplets^27,28^. To minimize this problem, O-dichlorobenzene was used to modify the ink composition, as it boils at 180 °C, which is higher than the boiling point (111 °C) of the previously used toluene. For every 10 ml of o-dichlorobenzene, 60 µl of toluene was added to the solvent mixture. Ultimately, all the inks produced contained a semiconductor dissolved in a solvent mixture (o-dichlorobenzene with toluene). The concentration of the organic semiconductor in the solvent mixture was 2 mg/ml.

To inkjet print the semiconductor layer, certain parameters were kept constant. The substrate temperature was 40 °C and the jetting frequency was 5 kHz. To deposit the semiconductor DPPDTT, a drop space of 20 µm was used and the number of the active nozzles was set to 6. To deposit the P3HT layer, up to 10 active nozzles were used and a drop space of 15 µm was applied. The layers printed with the developed inks exhibited smooth surfaces, with a slight accumulation of material at the center-to-bottom right of the layer, caused by the Marangoni effect (Fig. 2c)^29^. In the selected BGBC architecture, the semiconductor layer was positioned as the last layer at the very top. Since its surface therefore did not have to be perfectly flat, no further attempts were made to compensate the unfavorable flow of ink inside the droplet. The thickness of the semiconductor was around 100 nm on the edges and 200 nm at the center. Charge transport occurs only in the first few nanometers of a film, so differences in this range have no impact on charge transport^30^.

### Resistor and transistor

**Figure 3:**
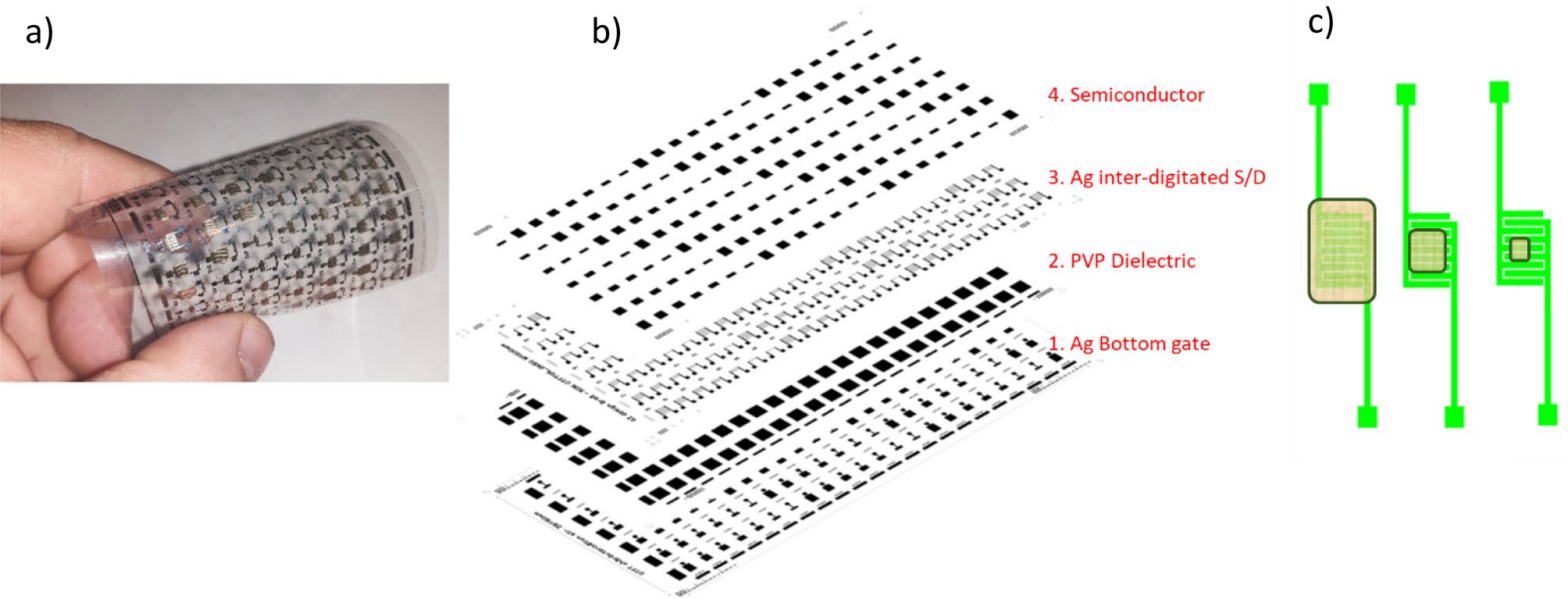
Photo of the elastic matrix of 154 printed transistors (**a**); images of the subsequent layers: Ag gate electrodes, PVP dielectric, Ag inter-digitated S/D electrodes, semiconductor DPPDTT (**b**). Scheme of the printed resistors with a P3HT layer, with different resistances resulting from varied layer geometry. From the left side—small, medium, and high resistance values (**c**).

Figure 3a shows a photo of an exemplary matrix of 154 printed transistors on an elastic polymer substrate. The differences between the OTFTs within the array results from their size i.e., the number of inter-comb source and drain electrodes, and hence the width (*W*) to length (*L*) ratio of transistor channel (*W*/*L* ratio). Dimensions of the printed electrodes and transistors channel, were measured using optical microscopy. Figure 3b shows a schematic arrangement of the individual printed layers. When printing the inter-digitated S/D patterns, it was very important to fix an optimal channel length, which serves as the both functional area for the OTFTs and as the active zone of the resistor.

For channel length *L* = 250 µm, the deviation was ± 15 µm, which is very typical for inkjet printing as the ink possesses low viscosity and the substrate’s surface energy is relative high.

**Figure 4:**
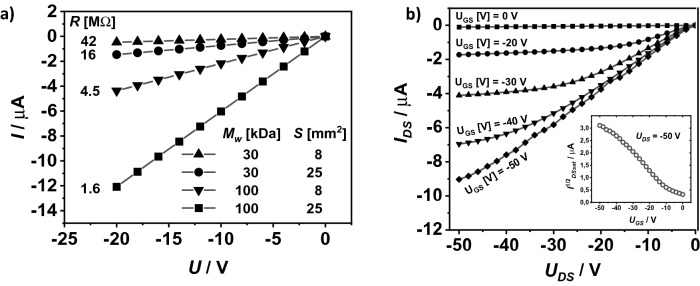
Electrical current–voltage output characteristic of the printed resistors (**a**), and characteristic of the printed transistor (the inset shows the dependence of the square root of the drain current on a gate-drain voltage) (**b**).

All-inkjet printed organic resistors were fabricated with the same inter-digitated electrodes that were used in the transistors, printed directly on the PEN substrate. The active layer of the resistors was made of the P3HT semiconductor. The printed resistors were kept in an ambient and daylight atmosphere for several days. Initially, the shape of the measured DC characteristics was non-linear and changed slowly, but after one week the process stopped. The characteristics reached an ohmic shape with a time-stable slope (Fig. 4a), caused by photo-induced oxidation of the P3HT^31^. The resistance calculated as a reciprocal of the slope of this line was dependent not only on the area of the P3HT layer (Fig. 3c), but also on the molecular weight of the polymer. This enabled the fabrication of a resistor with the desired resistance.

The output characteristics of an all-inkjet-printed OTFT made used DPPDTT and the dependence of the square root of a drain current on its gate-drain voltage are presented in Fig. 4b. The electrical parameters extracted from these characteristics are as follows: charge carrier mobility of 0.2 cm^2^V^−1^ s^−1^, threshold voltage of − 9 V, On/Off ratio of around 10^4^. These electrical parameters are comparable with those obtained for OTFTs made by spin-coating a semiconductor with a thermally evaporated gold source and drain electrodes, and are better than in the case of recently reported all-inkjet-printed OTFTs^19,32,33^.

The printing conditions were optimized to obtain resistor-transistor pairs that were characterized by the smallest possible voltage ranges Δ*U*_*0*_ and Δ*U*_*1*_ (see Fig. 1b). Small values for Δ*U*_*0*_ occurred in transistors with low zero current (*I*_*DSmin*_ <  < *I*_*DSmax*_). However, in order for Δ*U*_*1*_ to be as small as possible, it was necessary to create resistor-transistor pairs in which the channel resistance of the transistor operating in the linear region (the beginning of the output current–voltage characteristic of the transistor) was much lower than the resistance of the resistor (*I*_*DSmax*_ ~ *U/R*).

### Reference inverter

The inverters were analyzed in various combinations of manufactured resistors and transistors. Promising results were obtained for a transistor with a channel length of 150 µm and width of 30 mm. The resistor was printed with electrodes of the same dimensions.

**Figure 5:**
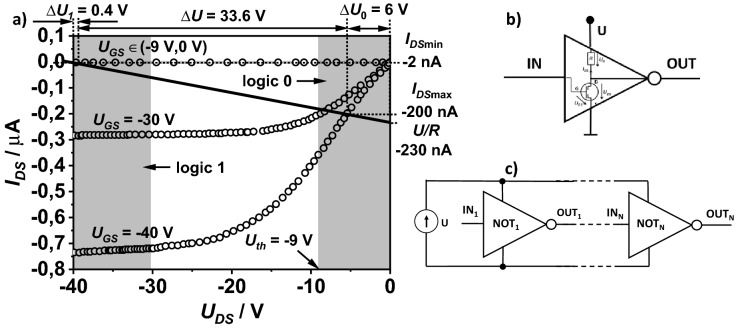
Output curves of the transistor with the load line (straight black line). The voltages Δ*U*, Δ*U*_*1*_, and Δ*U*_*2*_ are marked under the upper scale. The ranges of the logic states are marked as gray areas. On the right scale, the maximum and minimum currents are marked (**a**). Graphical symbol of the NOT logic gate (**b**). Serial connection of NOT logic gates (**c**).

The printed P3HT layer had an area of 0.5 mm^2^ and the molecular weight of the polymer was 100 kDa^34^. The resistance calculated on the basis of the current–voltage characteristic of the resistor was 170 MΩ, and the *U/R* ratio was − 2.35 10^−7^ A for *U* =  − 40 V. Based on these parameters, the load line was obtained for the reference inverter, designed with separately selected printed elements (Fig. 5).

The values of the currents *I*_*DSmin*_ and *I*_*DSmax*_ and the value of the voltages Δ*U*_*1*_ = 0.4 V and Δ*U*_*0*_ = 6 V were taken from Fig. 5a, and from Eq. (2) we obtained *U*_*OUT,1*_ =  − 39.6 V and *U*_*OUT,0*_ =  − 6 V. The inverter was characterized by good performance. For gate-source voltage *U*_*GS*_ ϵ(*U*_*th*_,0 V), *U*_*DS*_ did not exceed − 39 V. When *U*_*GS*_ ϵ(− 40 V, − 30 V), the *U*_*DS*_ was in the range (*U*_*th*_, 0 V). We assume that a *U*_*th*_ range from − 9 to 0 V is a logical zero, and the voltage range from − 40 to − 30 V corresponds to a logical one. The lower value of the limit voltage for a logical one (− 40 V) is the supply voltage *U* of the system. The upper limit results from the intersection of the load line for the voltage *U*_*th*_ with the output characteristic of the transistor at *U*_*GS*_ =  − 30 V. In this case, a reference inverter with the current–voltage characteristics shown in Fig. 5a will work as a NOT logic gate. The symbol of this a gate is shown in Fig. 5b. The key properties of the gate are summarized in Table 1.

**Table 1:** Truth table for the modeled inverter.

*U*_*IN*_ [V]	Logic	*U*_*OUT*_ [V]	Logic	Δ*U*/*U*	*I*_*DSmax*_/*I*_*DSmin*_
− 40 < *U*_*GS*_ < − 30	1	− 9 < *U*_*DS*_ < 0	0	0.84	110
− 9 < *U*_*GS*_ < 0	0	− 40 < *U*_*DS*_ < − 39	1		

A positive feature was the presence of a threshold voltage in the transistor for which the inequality |*U*_*th*_| > Δ*U*_*1*_ occurred. This ensured stable operation of the system, because after applying voltage to the input in the range from − 40 to − 39 V the voltage at the gate output was − 6 V. After applying the voltage to the input in the range from − 9 to 0 V at the output, we obtained − 40 V < *U*_*DS*_ <  − 39 V. Both values of the output voltages were far from the limit values of the logical states.

The considered model of the NOT gate was characterized by resistance to disturbances resulting from accidental voltage changes. This was because the voltage difference between the zero and logical one level was over 80% of the supply voltage value (Δ*U*/*U* = 0.84). The relatively large value of the factor *I*_*DSmax*_/*I*_*DSmin*_ also protects the device against interference from random noise causing accidental current fluctuations in the output circuit. In relation to *I*_*DSmin*_, the transistor was characterized by a small leakage current (less than 10^−10^ A). This facilitated cascading of the devices, since the input current of the NOT gate does not cause a significant change in the current flow in the output circuit of the preceding NOT gates. It should be noted that the possibility of connecting NOT gates is desirable when building most logic circuits (for example, a ring oscillator^6,35^).

The model NOT gate described in this work was not constructed in reality. Only the theoretical operation of the device was analyzed. Based on the satisfactory results, it was decided that the ink formulas, geometry of the electrodes, and parameters of the printing process used to create the reference inverter would be applied in fully printed inverters.

### Fully printed inverter

**Figure 6:**
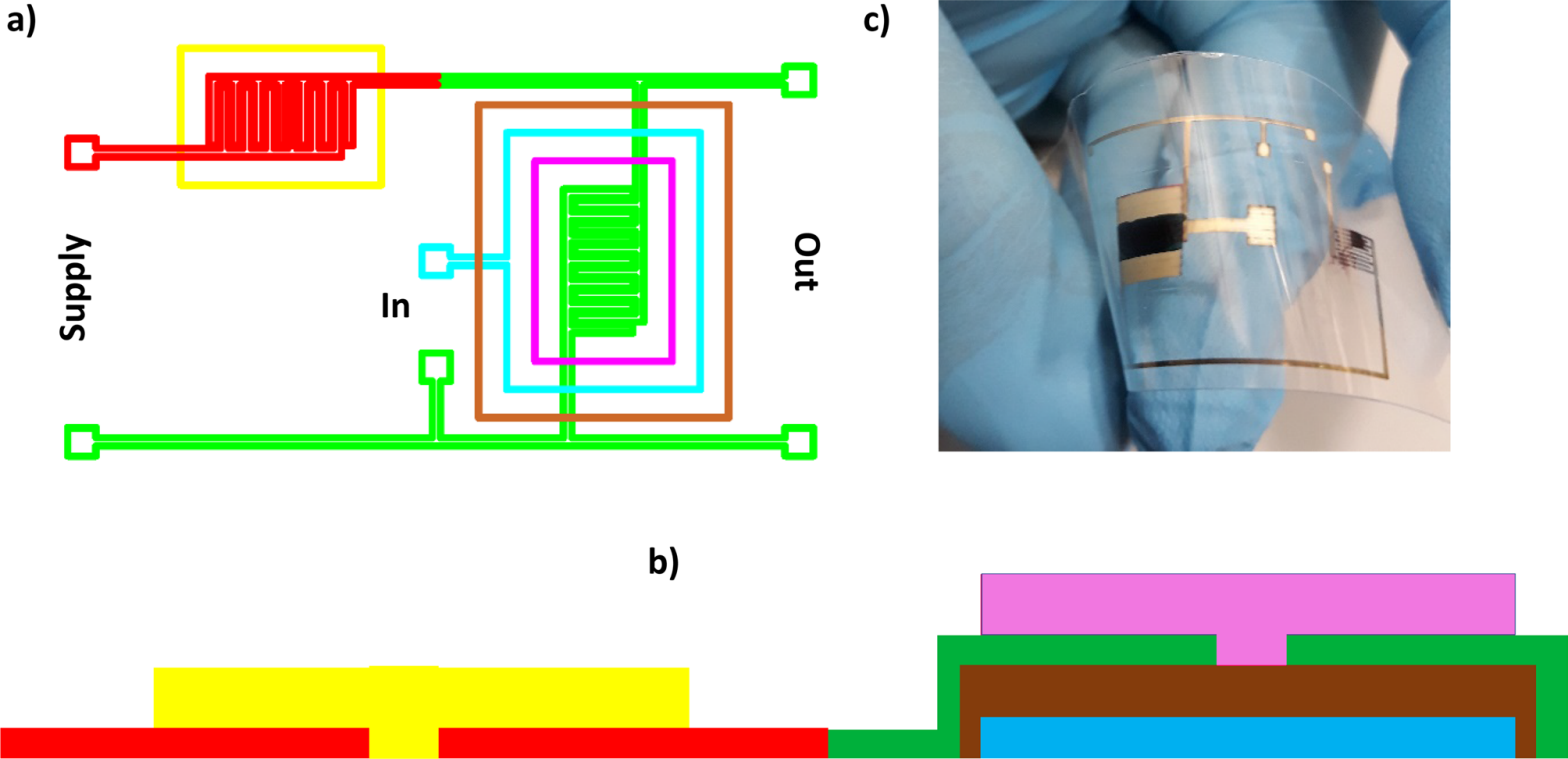
Schematic diagram of the NOT gate, showing successive printed layers: the gate electrode (blue rectangle area); dielectric printed on the electrode surface (brown rectangle area); source and drain electrodes printed on the dielectric with conducting paths (green color); printed resistor comb electrodes (red color): semiconductor printed as the last layers of the DPPDTT (pink rectangle area); P3HT doped with water and oxygen (yellow rectangle area) (**a**,**b**); Photo of the printed inverter. The resistor is located on the right-hand side and the transistor on the left-hand side of the image (**c**).

Figure 6a,b shows a six-layer stack of the printed inverters. The transistor and resistor were printed in the same configuration as the separate devices. In the resistor part, the area and molecular weight of the P3HT were matched to the characteristics of the transistor. The channel width of the transistor was adjusted by changing the dimensions of the inter-comb source and drain electrodes.

Figure 6c shows a photograph of the printed device. The P3HT layer (resistor) has an area of 4 mm^2^. The DPPDTT layer and interdigitated electrodes form a transistor channel with a width of 50 mm and a length of 150 µm.

The same procedure was used to produce the fully printed gates as for the production of the reference inverter. However, different results were obtained. Currents *I*_*DSmin*_ and *I*_*DSmax*_ and the threshold voltage *U*_*th*_, were unexpectedly different from those when the devices were printed separately. This was due to the additional printing and annealing processes after printing the organic semiconductor. The already deposited semiconductor layer was subjected to solvent vapor annealing, which changed its morphology and influenced the parameters of the organic transistors. This effect was visible as a change in the color of the P3HT layer during printing of the DPPDTT film. Despite the worse parameters of the transistors and resistors, it was possible to print inverters that were suitable for the construction of NOT gates. An analysis of the operation of these devices is presented below.

We selected the two inverters (INV1 and INV2) with the best parameters of resistor characterized by relatively high resistance compared to the channel resistance of the transistor operating in the linear voltage range (low value Δ*U*_*0*_). The current–voltage characteristics of the transistors and resistors were detected separately (Fig. S3 in the supplementary materials).

Resistors showed linear characteristics in the range of voltages needed for polarization of the inverters. For INV1 it was the range from − 50 to 0 V, and for INV2 from − 40 to 0 V. The resistor area and the transistor channel width in INV2 were much larger than in the transistor in INV1, which contributed to the threefold lower resistance of the resistor (*R*_*INV1*_ = 1.1 GΩ and *R*_*INV2*_ = 250 MΩ) and a drain current higher in INV2 than in INV1 (*I*_*DSmaxNV2*_ = 156 nA and *R*_*DSmaxINV2*_ = 38 nA). Due to the fulfillment of the condition of a small value for Δ*U*_*0*_, the range of the drain current of the transistors in both devices did not exceed 200 nA (gray rectangles on the output characteristics of the transistors (Fig. S3a,b). The transistor in INV1 had a threshold voltage of − 9 V. In INV2, the threshold voltage in the transistor was 0 V, and the channel resistance was much lower in the “off state” than in the transistor in INV1 (the lower the channel resistance in the off state, the more significantly the transistor off-current increases with higher *U*_*DS*_ voltage).

**Figure 7:**
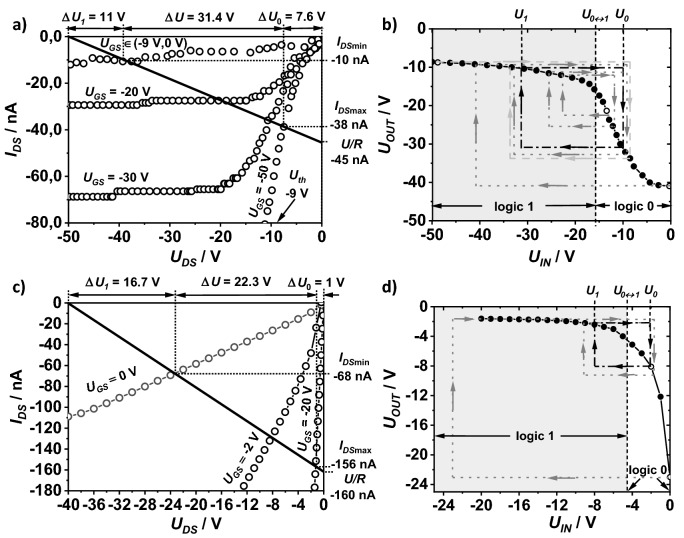
Load line for the fully-printed inverter INV1 with a transistor with a layer of DPPDTT and a resistor with a layer of P3HT, molecular weight 30 kDa, area 2 mm^2^, supplied by − 50 V (**a**); and for INV2 where the drain current was enhanced in relation to INV1 by increasing the channel width of the transistor and the dimensions of the P3HT resistor to 6 mm^2^ (**c**). Transfer characteristics of INV1 (**b**) and INV2 (**d**). Gray dashed lines with arrows indicate the directions of changes in the input and output voltages of successive inverters. The light gray lines and arrows show the voltage evolution after applying the smallest voltage in the circuit (logical 1) to the input of the first inverter. The dark gray lines and arrows show the voltage evolution after applying a voltage higher than the threshold voltage U_0↔1_ (logical zero). The black dashed-dotted lines and black arrows show a closed cycle of voltages on the inputs and outputs of three (or more) inverters connected in series (**b**,**d**).

Figure 7a,c show load lines plotted with the current–voltage characteristics of the transistors for both inverters which the voltage ranges of the transistor Δ*U*_*0*_ were determined. They were much lower than the voltage ranges Δ*U*_*1*_. This was a result of the relatively large values of the zero currents and the low resistance of the transistor channels in the linear range. The voltage ranges Δ*U*_*1*_ = 11 V for INV1 and Δ*U*_*1*_ = 16.7 V for INV2 were lower than the absolute values of the threshold voltage |*U*_*th*_|. The Δ*U*/*U* ratios 0.64 and 0.54, as well as the *I*_*DSmax*_/*I*_*DSmin*_ current ratios 3.8and 2.8 for INV1 and INV2, respectively, were less favorable than for the reference inverter (see Table 1).

Figure 7b,d show the transfer characteristics *U*_*OUT*_(*U*_*IN*_) of the fabricated inverters.

The *U*_*0↔1*_ threshold voltages of the two inverters were determined that corresponded to the condition of equalizing the output voltage and the input voltage of the inverter (*U*_*OUT*_ = *U*_*IN*_). The voltage *U*_*0↔1*_ corresponds to the limit value of the input and output voltages of the NOT gate, above and below which we have to deal with the logic states zero and one, respectively. Applying the voltage *U*_*IN*_ > *U*_*0↔1*_ to the input of the system caused the appearance of the voltage *U*_*OUT*_ < *U*_*0↔1*_ on the output. Conversely, when *U*_*IN*_ < *U*_*0↔1*_ the voltage *U*_*OUT*_ < *U*_*0↔1*_ was registered at the output.

**Figure 8:**
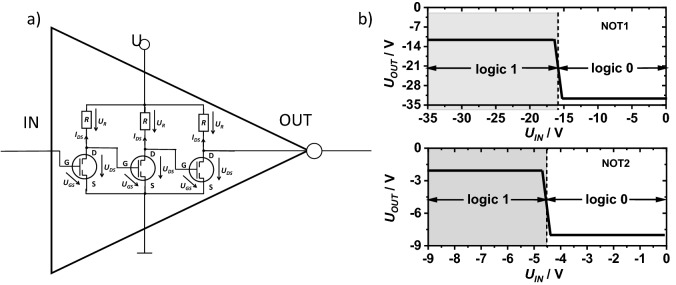
Electrical scheme of the NOT logic gate with three printed inverters connected in series (**a**) and predicted transfer characteristics of the gate constructed using three identical inverters: INV1 (up) or INV2 (down) (**b**).

In the case of several cascaded interconnected inverters, as shown in Fig. 5c, the input and output voltage levels in consecutive devices approach the set values of *U*_*0*_ and *U*_*1*_ as the number of interconnected inverters increases (trace the gray dashed lines and arrows in Fig. 7b,d). For example, consider inverters connected in a series with electrical properties similar to INV1. Applying 0 V to the input of the first inverter causes − 41 V to appear on its output, which will also be the input voltage for the second inverter. A voltage of − 9.2 V will appear at the output of the second inverter, and the input of the third inverter, activated with this voltage, will give the output a voltage of about − 31 V. At the output of the next inverter, the voltage will be − 10 V. Adding more inverters does not change the voltage states much (the black dotted-dashed lines and arrows in Fig. 7b). The voltages *U*_*0*_ and *U*_*1*_ on the input and output of every second inverter will be the same. It will also be noticed that the voltage *U*_*0*_ at the output of three or more interconnected inverters will appear with any input voltage in the range from − 50 to − 16 V (logical one). Similarly, the voltage *U*_*1*_ appears when the input voltage is in the range from − 16 to 0 V (logical zero). Consequently, it is enough to connect at least 3 inverters in series to obtain a NOT gate with well-defined logical states. Figure 8a shows a schematic diagram of a gate consisting of three inverters connected in series. The transfer characteristics of the NOT1 and NOT2 gates are shown in Fig. 8b. Their parameters are summarized in Table 2.

**Table 2:** Truth table for the inverters.

Gate	*U*_*IN*_ [V]	Logic	*U*_*OUT*_ [V]	Logic	*U* [V]
NOT1	− 50 < *U*_*GS*_ − 16	1	*U*_*DS*_ = − 10	0	− 50
	− 16 < *U*_*GS*_ < 0	0	*U*_*DS*_ = − 31	1	
NOT2	− 40 < *U*_*GS*_ < -4.5	1	*U*_*DS*_ = − 2.1	0	− 40
	− 4.5 < *U*_*GS*_ < 0	0	*U*_*DS*_ = − 8	1	

Analysis of the production and performance of the fully printed NOT gates containing three or more serially connected inverters is beyond the scope of the present study. However, if three or four inverters are printed close to each other, there is a strong chance that the resulting NOT gate will work properly.

Another very important issue is the long-term stability of devices operating under ambient conditions. A potential solution is to encapsulate the devices, which would prevent degradation of the semiconductors and electrodes by oxygen and water. The printed resistors, transistors, and inverters maintained their electrical properties, despite the fact that they were fabricated under ambient conditions and not encapsulated. Figure S4 in the supporting materials shows the current–voltage characteristics of the printed resistor and transistor immediately after their manufacture (Fig. S4a) and after three years of storage in air, at room temperature (Fig. S4b). The characteristics of the devices do not show significant changes. Only the zero current of the transistor increased. Analyzing the electrical properties of the reference inverter and the inverters INV1 and INV2 shown in Figs. 5 and 7, it can be seen that the zero current of the transistor has a key influence on the properties of the inverters. The higher the current, the worse the parameters of the devices. It is worth noting that the zero current of the transistor stored for three years decreased significantly after heating in an oven at 150 °C (Fig. S4c). The other properties of the resistor and the transistor did not change, indicating that annealing effectively removed the water from the semiconductor film, preventing the water doping effect. Therefore, the printed inverters do not have to be encapsulated, but only regenerated from time to time at an increased temperature. Encapsulation could even be disadvantageous, because as water and oxygen molecules slowly penetrated the barrier they would also be impeded from escaping the structure. Eventually, the device would be inoperable because the water and oxygen would prevent temperature regeneration.

## Conclusion

This paper has presented a method of manufacturing voltage inverters that can be used as components in fully printed NOT logic gates. The devices were printed with ink containing conductors (silver nanoparticles), organic semiconductors (DPPDTT and P3HT), and an insulator (PVPh). The process of printing organic transistors and resistors separately on different flexible PEN foils was found to be highly repeatable. The process of printing inverters on one PEN sheet, where the transistor and resistors were connected by a printed conductive path, was much more complicated than printing the separate devices. The organic semiconductors were very sensitive to solvents. Printing the semiconductor on top of another semiconductor caused solvent vapor contact with the printed film. In the annealing process, the solvent vapor changed the morphology of the already printed semiconductor. In the case of P3HT, crystallization of the macromolecules caused an increase in the conductivity of the film. After contact with the solvent vapor, the DPPDTT film was dopped by oxygen molecules, which caused an increase in the transistor off current and shifted the threshold voltage toward positive values. However, these difficulties were overcome and working voltage inverters were printed. It was not possible to build a gate with a single inverter.

The results obtained for a single inverter allowed us to analyze the behavior of more than one inverter connected in series to form a NOT logic gate. Theoretical analysis of a series of the same inverters showed that when three inverters were connected the logic states were precisely defined. This enables the creation of complex electronic devices, based on the described inverters.

For practical applications, large numbers of interconnected gates with similar electrical properties should be printed on large surfaces. This is much more difficult than printing single inverters and requires additional research and testing. The main challenge is to determine the acceptable morphological differences when printing is carried out under normal atmospheric conditions. The printing technology should be developed in such a way that the differences between the parameters of the NOT gates do not exceed the established ranges.

## Experimental section

### Materials and equipment

A semiconductor Poly[2,5-(2-octyldodecyl)-3,6-diketopyrrolopyrrole-alt-5,5-(2,5-di(thien-2-yl)thieno [3,2-b]thiophene)] DPPDTT with Mw = 290,668 kg/mol and polydispersity index PDI = 2.03, poly(3-hexylthiophene-2,5-diyl) P3HT with Mw = 94,100 kg/mol and polydispersity index PDI = 1.90 and with Mw = 34,100 kg/mol and polydispersity index PDI = 1.75 was purchased from Ossila company. The solvent ethyl lactate with HPLC grade, polymer poly (4-vinylphenol) (PVPh), and cross-linking agent poly (melamine-co-formaldehyde) (PMF) were procured from Sigma-Aldrich.

All these compounds and solvents were used as received, without additional treatment. The nano-particle-based silver ink UTDAgIJ was purchased from UT Dots Inc. An acrylic coated 125-µm thick polyethylene naphthalate (PEN) based Teonex® substrate Q65FA was bought from DuPont Teijin Films. To facilitate the deposition of the functional layers, a Drop-on-Demand (piezoelectric actuation) lab-scale printer was used (Dimatix Material Printer DMP-2831 from Fujifilm Dimatix Inc. with 10 pL based DMC printheads). The printheads offer up to 3 mL of ink carrying capability, along with variable printing resolution—i.e. 5 µm (5080 dots per inch) to 254 µm (100 dots per inch) drop spacings.

### Printing the individual layers of the device

Electrodes and conductive pads were printed using UTDAgIJ nano-particle based silver ink. The average size of the silver nanoparticles was around 10 nm, yielding a high conductivity of 10^5^ S/cm^36^. The piezoelectric printhead was tuned to a 10 pL DMC cartridge. The sintering temperature for the PEN Teonex® Q65FA substrate film was set to 150 °C. To print the dielectric layer, a jettable ink was formulated with 0.9 g of PVPh polymer and 0.78 g of PMF cross-linker dissolved in 70 ml of ethyl lactate. The ink was prepared in the laboratory, just before the printing process. For deposition of the dielectric layer, the following printing parameters were implemented: 25 µm drop space (digital printing resolution 1016 dpi); up to 10 actively printing nozzles; 1 mm printhead to substrate distance; 5 kHz jetting printing frequency; substrate at room temperature. A polymeric p-type semiconductor, Poly[2,5-(2-octyldodecyl)-3,6-diketopyrrolopyrrole-alt-5,5-(2,5-di(thien-2-yl)thieno [3,2-b]thiophene)] (DPPDTT), was selected to print the active layer in the organic transistor. The printable ink contained DPPDTT with a concentration of 2 mg/ml, dissolved in a combination of 1,2-dichlorobenzene and toluene in 1/0.006 proportion. The addition of a small amount of toluene to the ink caused a reduction in the coffee stain effect (Fig. S1 in the supplementary materials), due to an increase in Marangoni flow in the drying film^37^.

### Printing the resistors and transistors

The OTFTs were printed as multilayers stacked on polymeric PEN substrate in the BGBC configuration. First, a silver (Ag) gate electrode was printed on PEN Teonex® Q65FA. The foil was then placed inside a convection oven, where the printed layer was sintered at 150 °C for 20 min. The PEN substrate along with the conductive pads were again moved to the inkjet printer, and two consecutive layers of PVP dielectric were printed (wet-on-wet). The dielectric was again thermally cured in the oven at 150 °C for 45 min. The source (S) and drain (D) electrodes were printed on the cured dielectric layer using the same Ag ink, and finally subjected to a sintering process at a temperature of 150 °C for 30 min. Finally, the DPPDTT was printed as a semiconductor film and the device was annealed at 80 °C for 20 min. The resistors were printed in a similar way to the transistors, but without the deposition of the gate electrode and dielectric layers.

### Characterization

The device was characterized under ambient conditions, using a Keithley 2634B Source Meter connected to a needle-probe station. A schematic of the electrical circuit, including the tested voltage inverter and the source-meter instruments, is shown in Fig. 1a. The transfer and output characteristics of the OTFTs were measured within a voltage regime from 0 to − 70 V. The mobility of charge carriers (*µ*_*FET*_) and the threshold voltage (*U*_*th*_) were determined by a standard method based on analysis of the dependence of the square root of the drain current (*I*_*DSsat*_) on the voltage prevailing between the gate and the source (*U*_*GS*_) in the saturation regime^38^. The topographical scans of the printed layers and their surfaces were characterized using a Nanosurf Flex atomic force microscope. The data were processed using Gwyddion software^39^.

## Supplementary Information

Supplementary Information.

## Data Availability

The data generated during the experiments are available from the corresponding author on reasonable request.
