# Supplementary material for: Fully inkjet-printed flexible organic voltage inverters as a basic component in digital NOT gates
Source: Sci Rep. 2022 Jun 28;12:10887. doi: 10.1038/s41598-022-14797-4 (PMC9240068; doi:10.1038/s41598-022-14797-4)
Supplement: Supplementary file 1 — Supplementary Information. [file 41598_2022_14797_MOESM1_ESM.pdf]

Supporting Information

Fully inkjet-printed flexible organic voltage inverters as a basic component in digital NOT gates

Adam Luczak, Kalyan Y. Mitra, Reinhard R. Baumann, Ralf Zichner, Beata Luszczynska, and Jaroslaw Jung*

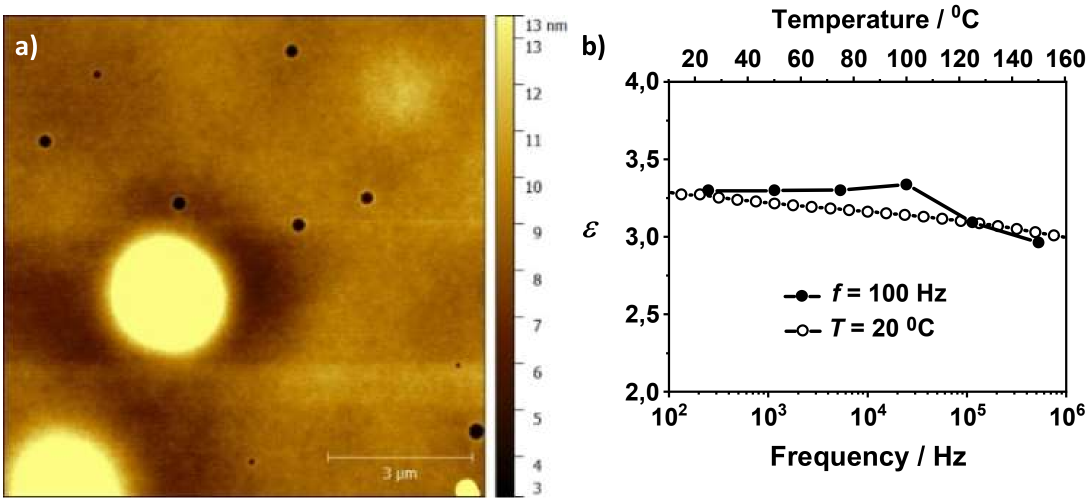

Figure S1. AFM images of the surface of a printed PVPh dielectric film a). Yellow round areas are agglomerations of undissolved PVPh, and black dots are the holes in the film. Dielectric permittivity as a function of frequency (white circles) and of temperature (black circles) measured for the printed dielectric film b).

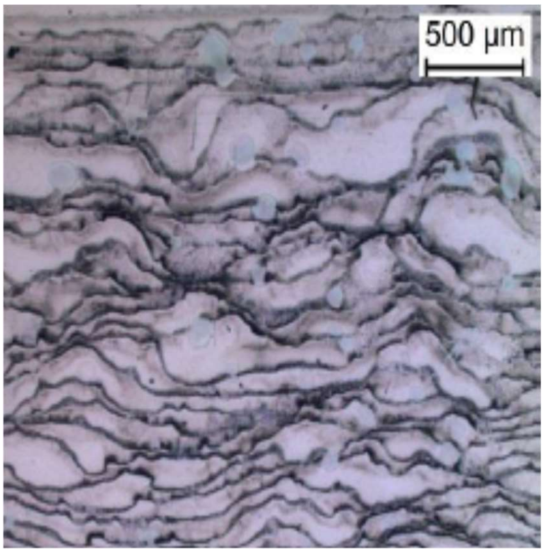

Figure S2. Microscopic image of the surface structure of a DPPDTT layer printed using toluene based ink. The coffee-stain effect is visible on the dry edges of the ink drops.

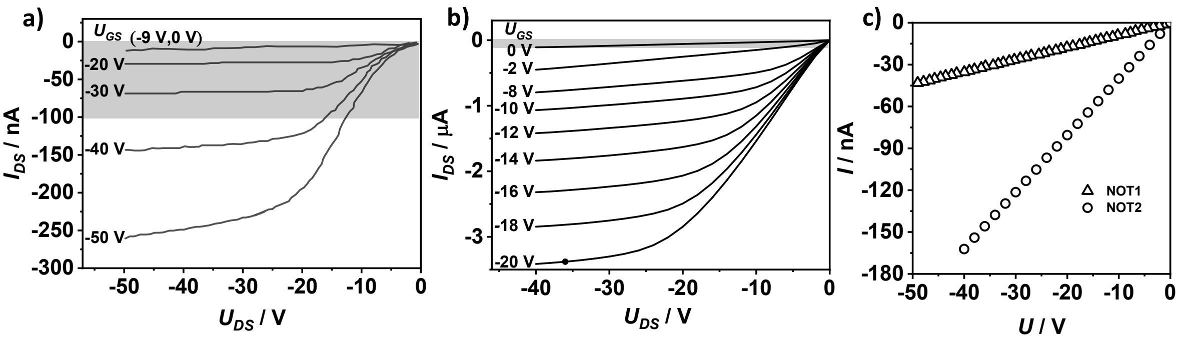

Figure S3. Electrical current-voltage output characteristic of transistors with a DPPDTT layer. The transistors were components in INV1 a) and INV b) electrical circuits. The current-voltage operating ranges of the inverters are marked by gray rectangles. Current-voltage characteristics of the resistors with a P3HT layer for INV1 and INV2 electrical circuits c).

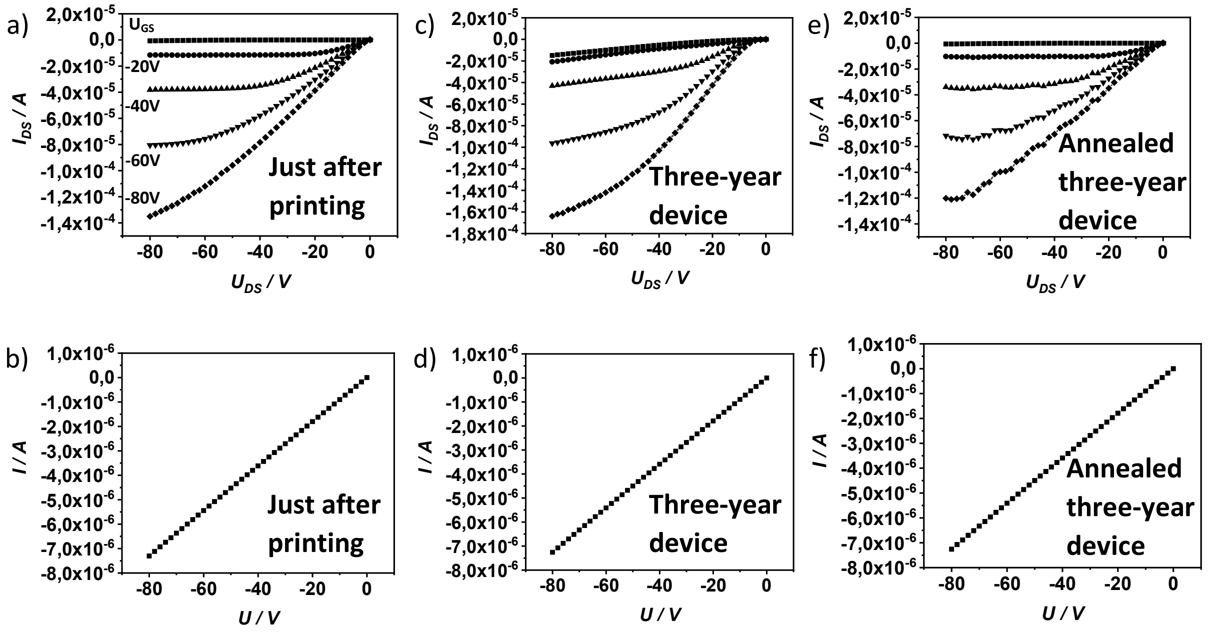

Figure S4. Electrical current-voltage characteristics of the transistor and resistors measured: just after printing a) and b); three years after printing c) and d); after annealing of three-year old devices e) and f).

Optimization of printing process

In all printing process we started from standard printing parameters: The substrate temperature of 40 °C, a drop space of 20 µm and the jetting frequency 5 kHz and 8 active nozzles. The start parameters were changed to obtain the good quality of printed films.

Electrodes

In the Figure S5 we are showing the microscopic images of printed silver electrodes.

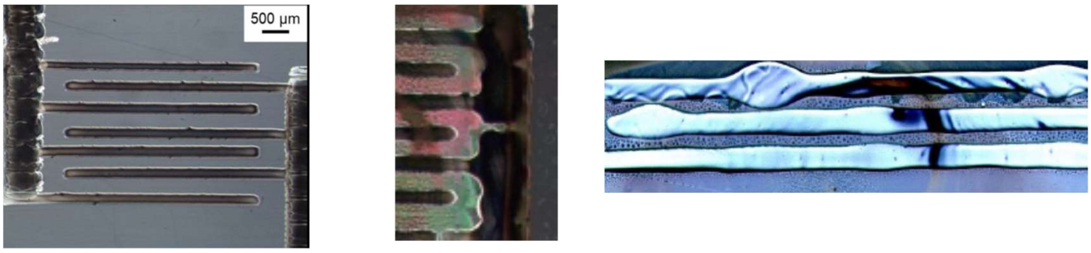

Figure S5. Microscopic images of a) printed electrodes and b) and c) examples of defects.

Optimization process was staring from adjusting the temperature of substrate. In higher temperature, spreading of the printed drop was sharply limited. At this step, the temperature substrate of 40°C was found as optimal.

In the next step, the waveform controlling the printing head was adjust, to obtain stable droplets, without any satellites and leaks. The optimized waveform is presented on the **Fig. S6**.

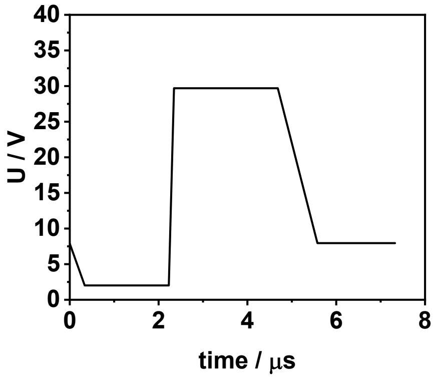

Figure S6. Waveform used in printing of the electrodes.

In the last step, the number of active nozzles and drop space were optimized to obtain stable printed pattern. In this case, the number of nozzles was reduced or/and the drop space was increased to remove the effect of "bubbles" (Fig. S5c). When the number of active nozzles was too low and/or the droplet space value was too high, the electrodes were discontinuous (Fig. S5b). To optimize the process, 3 active nozzles and a droplet space of 40µm were used.

Dielectric

The optimization process was the same as for electrode printing. During this process we encountered two problems with the printed foil substrates, described in the text of this article. We have dissolved these problems by printing two layers of PVPh using a wet-on-wet technique. Using this method, we change the dielectric surface from a rough one containing numerous defects (Fig. S1a) to a smooth one without defects (Fig. 2b).

During optimization of the printing process, we obtain the waveform which is presented in Fig. S6.

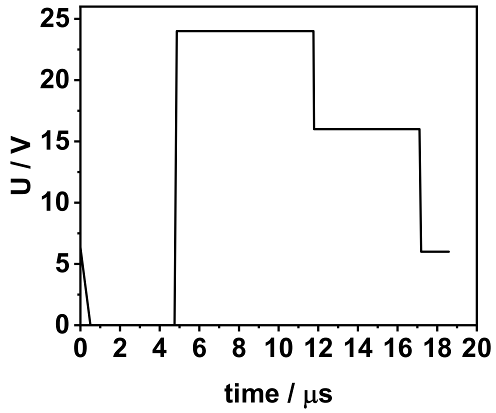

Figure S6. Waveform used in printing of the dielectric.

In consequence, we obtain the optimized printed parameters: drop-space – 25μm, substrate temperature – ambient, number of active nozzles – 10, jetting frequency – 5kHz.

Semiconductor

The optimization procedure was the same as before. The main problem with printing of semiconductor was the ink formulation. Optimization of the ink formula is described in the main text of article.

During the printing optimization, we have found the waveforms for P3HT and DPPDTT presented in Fig. S7a and Fig. S7b respectively.

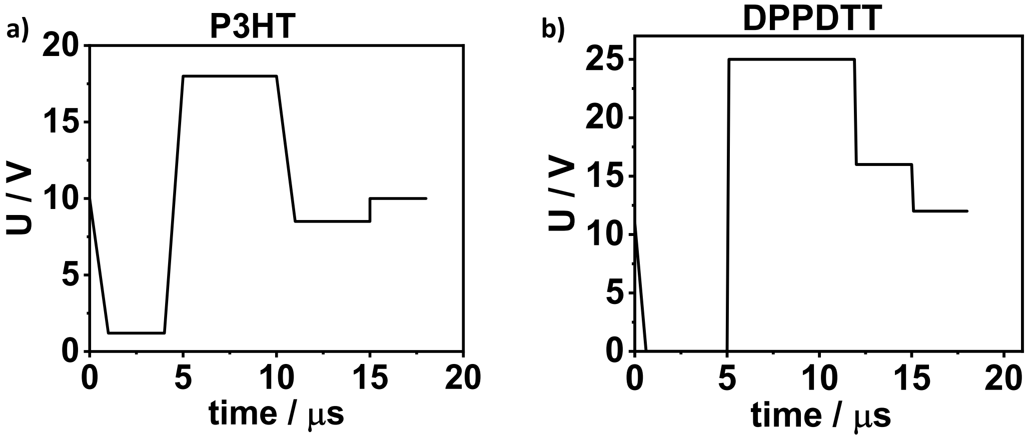

Figure S7. Waveform used in printing of the P3HT a) and DPPDTT b).

For printing process of semiconductors, we obtain the following optimized printed parameters:

- for P3HT: drop-space – 15μm, surface temperature – 40°C, number of active nozzles – 10, jetting frequency – 5kHz.
- for DPPDTT: drop-space – 20μm, surface temperature – 40°C, number of active nozzles – 6, jetting frequency – 5kHz.
